# Cerebral palsy and bruxism: Effects of botulinum toxin injections—A randomized controlled trial

**DOI:** 10.1002/cre2.207

**Published:** 2019-06-29

**Authors:** Birgitta Johansson Cahlin, Christopher Lindberg, Lars Dahlström

**Affiliations:** ^1^ Department of Orofacial Pain and Jaw Function and Mun‐H‐Center, National Orofacial Resource Center for Rare Diseases, Public Dental Service Region Västra Götaland, Institute of Odontology, The Sahlgrenska Academy University of Gothenburg Gothenburg Sweden; ^2^ Department of Neuroscience and Rehabilitation, Institute of Neuroscience and Physiology, The Sahlgrenska Academy University of Gothenburg Gothenburg Sweden; ^3^ Department of Behavioral and Community Dentistry, Institute of Odontology, The Sahlgrenska Academy University of Gothenburg Gothenburg Sweden

**Keywords:** botulinum toxin, bruxism, cerebral palsy, oral physiology, oral rehabilitation, TMD

## Abstract

**Objective:**

Cerebral palsy (CP) includes disturbances in muscular control caused by perinatal brain injury. Masticatory muscle involvement hampers functions such as chewing and talking. Bruxism and temporomandibular disorders are overrepresented. Neuromuscular blocks with botulinum toxin type A (BTX‐A) may alleviate problems due to muscular hyperactivity. The aim was to evaluate masticatory muscle BTX‐A injections in subjects with CP and bruxism.

**Methods:**

A prospective, parallel, randomized, placebo‐controlled, and double‐blind trial in 12 patients with CP was performed. End points were alterations in objective and subjective oral capacities after two BTX‐A or corresponding placebo injections. Matched, healthy references were also evaluated.

**Results:**

The reference group demonstrated stronger and more efficient oral functions compared with the CP group. Subjective and objective oral capacities appeared to vary considerably between CP patients and also over time in this patient group and were poorly correlated. No significant effect of BTX‐A compared with placebo on outcome variables was observed at group level, but continued treatment with BTX‐A was requested by the majority of the patients.

**Conclusion:**

The evidence is unable to support the use of BTX‐A for the treatment of affected masticatory muscles in CP, but the findings are inconclusive in certain respects. Larger, more homogeneous groups of CP patients need to be evaluated in future trials.

## 1 INTRODUCTION

Cerebral palsy (CP) is an umbrella term for different disturbances in muscular control caused by perinatal brain injury.(Koman, Smith, & Shilt, 2004; Richards & Malouin, 2013) This neurologic disorder occurs in two children per 1,000 births.(Himmelmann & Uvebrant, 2014) The spastic type is the most common, but there are also akinetic and dyskinetic types. When the masticatory muscles are affected, several important functions, such as chewing and talking, are hampered. Moreover, bruxism, the gnashing or grinding of teeth,(Lobbezoo et al., 2013) is often observed in individuals diagnosed with CP. Sleep‐related bruxism is reported in 8% of the general population,(Ohayon, Li, & Guilleminault, 2001) but it is strongly overrepresented, affecting up to 37–70%, among young CP patients.(Ortega, Guimaraes, Ciamponi, & Marie, 2007; Peres, Ribeiro, Juliano, Cesar, & Santos, 2007) This nonfunctional activity can last during both the day and night among subjects with CP and other dental patients with “special needs.” The utilization of neuroleptics in these categories is assumed to further aggravate the parafunction.(Lavigne, Kato, Kolta, & Sessle, 2003; Ortega, Dos Santos, Mendes, & Ciamponi, 2014) In general, bruxism is regarded as a risk factor for painful temporomandibular disorders (TMD).(Fernandes, Franco, Siqueira, Goncalves, & Camparis, 2012) The prevalence of TMD(Dworkin & LeResche, 1992) is considerable among young patients with CP, up to 68%,(Miamoto et al., 2011; Ortega, Guimaraes, Ciamponi, & Marie, 2008) as well as in patients with mental retardation.(Gurbuz, Kursoglu, Alatas, & Altinbas, 2010) Further, dental attrition/erosion, a possible consequence of spasticity/bruxism, is substantial in subjects with CP and significantly higher than in age‐matched controls.(Goncalves, Carmagnani, Correa, Duarte, & Santos, 2008) Reduced and altered saliva and frequent gastroesophageal reflux also play an important role in dental attrition/erosion.(Guare et al., 2012; Rodrigues Santos, Siqueira, & Nicolau, 2007)

As a result, subjects diagnosed with CP, as well as many other patients with “special needs,” can have refractory problems associated with neuromotor disturbances in the masticatory muscles, with serious consequences for oral function and dental structures. There is no proven effective therapy for the difficult clinical problem of spasticity/bruxism. Conventional treatment such as splints is seldom accepted, and daytime use might worsen already impaired speech.

Neuromuscular blocks with botulinum toxin have been used to treat spasticity in several different muscles in patients diagnosed with CP,(Tilton, 2015) and guidelines for the therapy of extremities have been compiled.(Olver, Esquenazi, Fung, Singer, & Ward, 2010; Sheean, Lannin, Turner‐Stokes, Rawicki, & Snow, 2010) The effect of botulinum toxin is reversible, and injections must be repeated. Botulinum toxin has also been used as a therapeutic alternative in painful maxillofacial conditions in otherwise healthy subjects(Mor, Tang, & Blitzer, 2015) and bruxism.(Long, Liao, Wang, Liao, & Lai, 2012; Manfredini, Ahlberg, Winocur, & Lobbezoo, 2015) The effect of local injections with botulinum toxin in chronic facial pain, associated with muscular hyperactivity and refractory to conventional treatment, has been reported to be significantly better than placebo,(Ihde & Konstantinovic, 2007) but its effect has also been questioned in controlled studies.(Ernberg, Hedenberg‐Magnusson, List, & Svensson, 2011; Nixdorf, Heo, & Major, 2002) Botulinum toxin type A (BTX‐A) has also been injected into the masticatory muscles to alleviate spasticity and bruxism in young patients with CP.(Manzano, Granero, Masiero, & dos Maria, 2004) The outcome of this uncontrolled pilot study was described as positive. Little is known about CP patients' own opinion of BTX‐A as a therapeutic alternative.

The aim of this study was to alleviate neuromotor disturbances in the masticatory muscles to improve function and reduce pain in subjects with CP and bruxism. Specifically, two injections with BTX‐A in musculus masseter and temporalis bilaterally were randomly compared with control conditions, saline injections. The outcomes, objective and subjective, were registered double blind. A matched, healthy reference group was also evaluated.

## 2 METHODS

The study design was prospective, parallel, randomized, placebo controlled, with double‐blind assessments.

### 2.1 Participants

Previous data on important outcome variables such as CP patients' own opinion of treating the masticatory muscles with BTX‐A were not available for power calculation. A significant improvement in masticatory muscle spasticity and bruxism after BTX‐A injections, as judged clinically, was reported in all six CP subjects included in a pilot study.(Manzano et al., 2004) Injections of BTX‐A in m. masseter can reduce the bite force from a mean of 51 kg/cm^2^ (standard deviation, *SD* 13) to 31 kg/cm^2^ (*SD* 12) (39%) in patients with masseter hypertrophy.(Ahn & Kim, 2007) A power analysis, based on this latter figure, gives a group size of eight subjects in each group (power 0.84, *P* < .05, and equal group size) to demonstrate effect on this variable. The intention was therefore to include 16 patients. Difficulties recruiting eligible patients in the region and the considerable inconvenience for the patients associated with all registrations resulted in cessation of the trial after 12 patients had been included.

The patients were recruited via hospital dental clinics in the Västra Götaland Region in Sweden. The inclusion criteria were man or woman, 18 years or older, diagnosed with CP and with reported bruxism, diurnal and/or nocturnal, also witnessed by relatives and/or caregivers, capable of making decisions and communicating without difficulty, and able to read and understand information. The exclusion criteria were an inability to understand the study and answer questionnaires. A minimum mental capacity was thus required. Further exclusion criteria were known sensitivity to botulinum toxin, infections in the injection area, pregnant or breastfeeding, ongoing treatment with botulinum toxin in other body parts, medication with aminoglykosid antibiotics, spektinomycin, or pharmaceuticals with a possible interaction with botulinum toxin.

Healthy volunteers, recruited from the staff but not in a dependency state, were matched for gender and age (± 5 years) against the first eight registered patients and constituted a reference group. They received financial compensation for their participation. All participants, patients and references, were examined and treated at the Mun‐H‐Center, a National Orofacial Resource Center for Rare Diseases in Gothenburg, from March 2013 to May 2015.

The Regional Ethical Review Board, University of Gothenburg, Göteborg, granted ethical approval for the study (dnr 870‐12). The Swedish Medical Products Agency also approved the study (dnr 15:2012/121788). Written informed consent was obtained from all participants.

### 2.2 Equipment, questionnaires

A bite fork with two legs, 76 mm long and 20 mm wide and linked at the base, was manufactured from stainless steel for the bite force measurements. The upper leg was 1 mm thick and the lower 2 mm for increased stability. A load button cell, 12.7 mm in diameter and 4 mm thick, was incorporated between the legs at the point of the fork. The total height of the bite fork was thus 7 mm. The bite fork was fixed to a 150 mm long handle. The function of the load cell, labeled FSH01068 (Futec Inc. USA), was based on the electrical resistance action of the strain gauges and was able to record force levels in the range of 0–1,100 N. The measured signals were transmitted via a cable to an electronic module, labeled LCV‐USB2 (Lorenz Messtechnik GmbH, Germany), where the analog signals from the load cell were amplified and then forwarded to an A/D circuit and converted to digital form. The recorded digital signals were sent via a USB cable to a PC, Dell Latitude E6410 with the XP operating system, and stored in Excel. The measured values were also shown on the screen in graphic form. The total system was calibrated over the entire measurement range against five known weights of 0 to 100 kg. Divergence varied between 0% and 0.3%. The bite fork was covered with a rubber tube to protect the teeth. The tube was marked to allow duplicate placement of the bite fork.

Chewing efficiency was evaluated using a color‐changeable chewing gum (Masticatory Performance Evaluating Gum XYLITOL®; Lotte Co. Ltd. Saitama, Japan). A color scale is used to assess the color change and is linked to a 0‐ to 100‐mm visual analog scale (VAS), so the results can be converted to a numerical value by measuring from the left‐hand end. Higher values imply more efficient chewing. Validity and reliability are acceptable.(Kamiyama, Kanazawa, Fujinami, & Minakuchi, 2010)

Questions about the participants' opinion of the “prevalence of bruxism,” “pain in the jaws,” “ability to chew,” and “ability to talk” during the preceding week were answered by marking on four 0‐ to 100‐mm VASs with the end points of “None/excellent” on the left and “Frequent/worst possible” on the right. The distance from the left‐hand anchor to the nearest mm of the marking was rated so that higher values implied more problems. The four questions were thought to have face validity. Moreover, all the participants answered a Swedish version of the General Oral Health Assessment Index (GOHAI)(Hagglin, Berggren, & Lundgren, 2005) with 12 Likert scale questions resulting in 12–60 scores, where higher scores imply more satisfaction with oral health status. The questionnaire has excellent reliability and validity.(Hagglin et al., 2005)

### 2.3 Procedure

**Figure 1:**
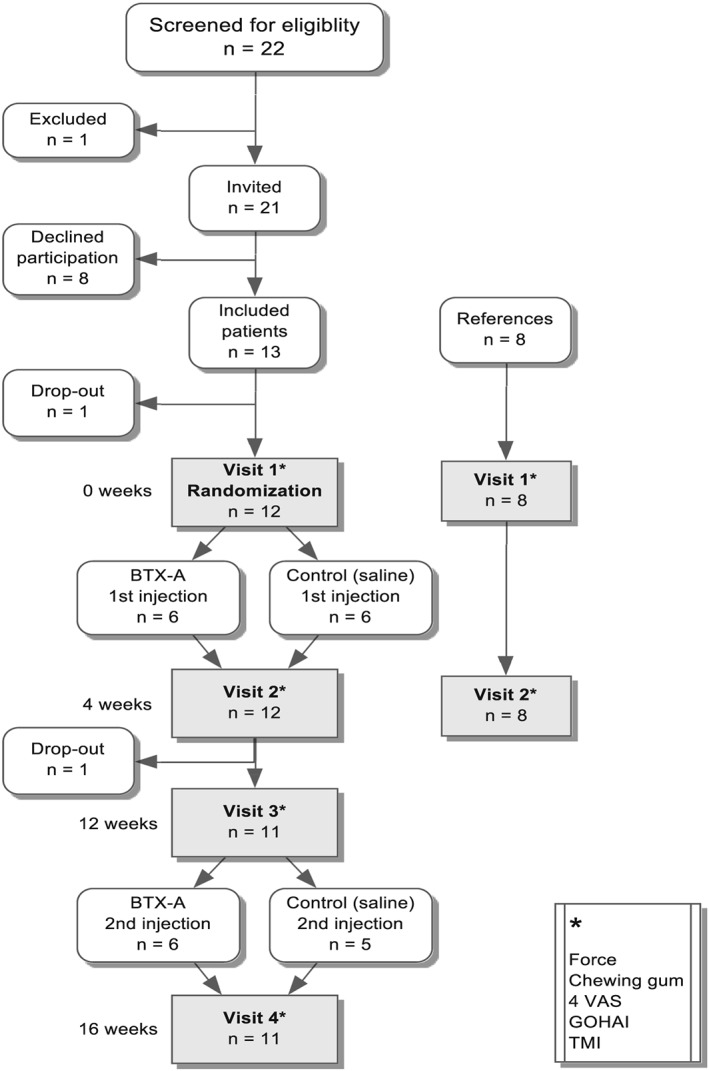
Protocol for the assignment of recruited participants to study groups and procedures. BTX‐A, blocks with botulinum toxin type A; GOHAI, General Oral Health Assessment Index; TMI, Temporomandibular Index; VAS, visual analog scale

A flow chart of the phases of the trial is shown in Figure 1. After the provision of informed consent, anamnestic data, including medication, were collected and orofacial examinations were performed by one orofacial pain specialist (B. J. C.) on the first (base) visit and noted in a case report form. Any diagnosis according to the Research Diagnostic Criteria for Temporomandibular Disorders (RDC/TMD) axis I (Dworkin & LeResche, 1992) was confirmed, and dental attrition/erosion was also registered.(Johansson, Haraldson, Omar, Kiliaridis, & Carlsson, 1993) The Temporomandibular Index (TMI)(Pehling et al., 2002) was used to quantify orofacial clinical signs and symptoms.

Bite force measurements were performed after calibration and familiarization with the equipment. The bite fork was placed in the same position at all registrations, distal to the right canine, and held by the operator whereas the force was registered without feedback. The verbal instruction was “Bite as hard as you can” for 3 s, and the peak value was registered. The mean of the three consecutive trials was noted, the maximum voluntary contraction (MVC). The instruction was then “Bite as you would when chewing an almond,” and the peak value was registered. The mean of five consecutive cycles was noted. Further, the maximum force between the index finger and thumb on the dominant hand was measured once and noted.

A standardized piece of room‐tempered, color‐changeable chewing gum was chewed for a total of 60 strokes. The chewing gum was then compressed in a polyethylene film to a thickness of 1.5 mm and photographed. Its color was rated directly by comparing it with the color scheme, and the corresponding numerical value on the linked VAS was determined and noted.

Finally, all the participants answered the four questions by ratings on the corresponding VASs and answered the GOHAI.

Half the patients were randomized to injections with BTX‐A and half to injections with saline after the registrations on the first visit. Randomization was performed via a table of random numbers by one person in the staff not involved in the trial. Numbered, opaque, closed envelopes were used in the allocation performed by the neurologist (C. L.) who administered all the injections, BTX‐A or saline. All patients received the allocated treatment.

The first injections were given after the registrations and randomization on the first visit at 0 week and the second after the registrations at the third visit at 12 weeks. Commercially available BTX‐A (Botox®, Allergan) or isotonic saline was given bilaterally in the masseter and temporal muscles. These muscles were chosen as powerful and easily accessible. The dose of BTX‐A, 100 units in 1.0‐ml saline solution, was distributed with 30 units in the masseter muscles and 20 units in the temporal muscles on each side. The injections were performed with electromyographic guidance (Injection Needle, 37 mm × 27G, Disposable Hypodermic Needle Electrode Luer Lock, CareFusion Germany 234 GmbH, Leibnizstrasse 7, 97204 Hoechberg, Germany). The batch number on the Botox® bottles was noted. Injections with isotonic saline solutions, 1.0 ml, were given in the same way at corresponding locations in patients randomized to control conditions. Care was taken not to reveal the content in the identical syringes to the patients or others. All data on injections were noted by the neurologist in journals, separated from the case report form and not available to other personnel.

All registrations, bite and finger–thumb force measurements, chewing gum recordings, and answers to the four questions on the VASs and GOHAI and the determination of the TMI, were made in precisely the same manner on all four visits at 0, 4, 12, and 16 weeks. One orofacial pain specialist (B. J. C.), blind to the treatment received, made all the observations. Questions about adverse events and side effects were asked on the second, third, and fourth visits. The reference group made two identical registrations of all 10 variables at 0 and 4 weeks.

The code was broken when all the patients and all the references had completed all the visits and all the procedures. All patients, still blinded, were offered BTX‐A injections 8 weeks after the last visit.

### 2.4 Statistical analysis

Statistical tests, nonparametric, were performed per protocol using a significance level of *p* ≤ .05, two‐tailed. For comparison between groups, the Mann‐Whitney *U* test was used for continuous variables, whereas Fisher's exact test was used to calculate the relationship between dichotomous variables. For comparison within groups, Wilcoxon's signed rank test was used. Spearman's rank correlation coefficients were also used for associations between objective and subjective variables at baseline within both patients and references.

## 3 RESULTS

Anthropometric and dental data and the distribution of RDC/TMD diagnoses for all participants, patients and references, are given in Table 1. Five of the patients medicated regularly: one with carbamazepine, one with diazepam, one with baclofen, one with lacosamide and levetiracetam, and one with citalopram. Only one reference subject used medication regularly, iron substitute.

**Table 1:** Distribution of anthropometric and dental data and temporomandibular disorder diagnoses (number of subjects fulfilling Research Diagnostic Criteria for Temporomandibular Disorders [RDC/TMD 0–III]) for all patients and references registered at the first visit

	BTX‐A patients n = 6	CTR patients n = 6	References n = 8
Gender, female (%)	50	33	25
Age years, mean (*SD*)	41 (13)	44 (17)	48 (17)
Number of teeth, mean (*SD*)	28 (5)	27 (2)	29 (2)
Occluding pair of teeth, mean (SD)	11 (5)	9 (1)	13 (2)
Dental attrition, mean (SD)	3 (1)	3 (1)	2 (1)
RDC/TMD 0, I, II, III	Ia (*n* = 2)	Ia (*n* = 1), Ib (*n* = 1)	Ia (*n* = 1)

Abbreviation: BTX‐A, blocks with botulinum toxin type A.

One patient reported side effects (“restless, tensed”) after the first injection (saline) at the second visit and decided to terminate the study prematurely. No other adverse events or side effects were reported or observed.

### 3.1 Comparison between patients and references

The results for all 10 outcome variables for the first eight registered patients at the first visit and the eight matched, healthy reference subjects at the first and second visits are given in Table 2. Statistically significant differences between patients and references were observed in five outcome variables, all in favor of the references. Among the references, the force at “Bite as you would when chewing an almond” increased to a statistically significant degree on the second registration. No other variable changed to a statistically significant degree over time among the references (see Table 2).

**Table 2:** Distributions of all 10 outcome variables, mean (SD)/median (range) for the first eight registered patients at the first visit (before randomization) and eight matched references at the first and second visits and p values for differences between patients and references (A) and between references at the first and second visits (B)

	Patients at first visit n = 8	A. p ≤ .05	References at first visit n = 8	B. p ≤ .05	References at second visit n = 8
Bite force, N, “as hard as you can,” MVC	242.0 (142.4)/198.3 (31.1; 458.0) *n* = 8	NS	327.9 (107.9)/273.5 (227.1; 484.6) *n* = 8	NS	390.4 (177.8)/303.1(246.4; 732.7) *n* = 8
Bite force, N, “as when chewing”	119.7 (99.9)/78.4 (17.1; 286) *n* = 8	NS	196.9 (124.6)/167.0 (34.7; 339.5) *n* = 8	0.02	277.1 (200.0)/208.6 (61.2; 576.0) *n* = 8
Finger–thumb force, N	18.0 (10.79)/16.2 (8.7; 30.9) *n* = 4	0.01	94.3 (27.4)/95.9 (54.5; 126.5) *n* = 7	NS	86.8 (20.6)/92.0 (57.2; 108.8) *n* = 7
Chewing efficiency, mm	28.8 (14.9)/25.0 (15.0; 50.0) *n* = 4	0.04	53.1 (16.7)/50.0 (30.0; 80.0) *n* = 8	NS	50.0 (15.8)/50.0 (30.0; 80.0) *n* = 8
VAS#1, mm “prevalence bruxism”	45.0 (32.9)/36.0 (10.0; 100.0) *n* = 7	NS	19.9 (27.4)/4.0 (0.0; 69.0) *n* = 8	NS	12.5 (22.1)/4.0 (0.0; 66.0) *n* = 8
VAS#2, mm “pain in the jaws”	18.8 (36.1)/0.0 (0.0; 97.0) *n* = 8	NS	6.0 (9.6)/1.5 (0.0; 22.0) *n* = 8	NS	5.6 (9.7)/2.0 (0.0; 28.0) *n* = 8
VAS#3, mm “ability to chew”	44.7 (20.7)/45.0 (12.0; 81.0) *n* = 7	0.00	1.5 (2.0)/0.5 (0.0; 5.0) *n* = 8	NS	2.1 (2.7)/1.5 (0.0; 8.0) *n* = 8
VAS#4, mm “ability to talk”	32.0 (39.1)/11.5 (0.0; 94.0) *n* = 8	NS	2.6 (3.3)/1.0 (0.0; 9.0) *n* = 8	NS	1.4 (1.6)/1.0 (0.0; 4.0) *n* = 8
GOHAI scores	42.1 (9.5)/43.5 (29.0; 56.0) *n* = 8	0.00	57.4 (3.5)/59.0 (50.0; 60.0) *n* = 8	NS	57.9 (2.6)/59.0 (53.0; 60.0) *n* = 8
TMI scores	0.10 (0.05)/0.08 (0.07; 0.21) *n* = 8	0.03	0.04 (0.06)/0.00 (0.00; 0.16) *n* = 8	NS	0.03 (0.04)/0.00 (0.00; 0.11) *n* = 8

Abbreviations: GOHAI, General Oral Health Assessment Index; MVC, maximum voluntary contraction; NS, not significant; TMI, Temporomandibular Index; VAS, visual analog scale.

Associations between objective variables (three force measurements, chewing efficiency, and TMI) and subjective variables (four VASs and GOHAI) were calculated among the first eight registered patients and among the eight matched references at the first visit. Among patients, good chewing efficiency was associated with high self‐rated “pain in the jaws” (*r*_s_ .88, *p* = .05) and with low self‐rated “ability to chew” (*r*_s_ .97, *p* = .01) and with low self‐rated oral health (*r*_s_ −.95, *p* = .01). These three correlations among patients differed to a statistically significant degree from the corresponding correlations among the references (all *p* = .01). Among the references, high self‐rated “pain in the jaws” was associated with low chewing efficiency (*r*_s_ −.75, *p* = .03) and with high bite force “as when chewing” (*r*_s_.70, *p* = .05).

### 3.2 Double‐blind comparison of BTX‐A and control treatment

There were no statistically significant differences between the active and control arms for any of the variables at any of the four registrations but one. The rating of “ability to chew” (VAS#3) was significantly better in the active arm on the second visit.

**Figure 2:**
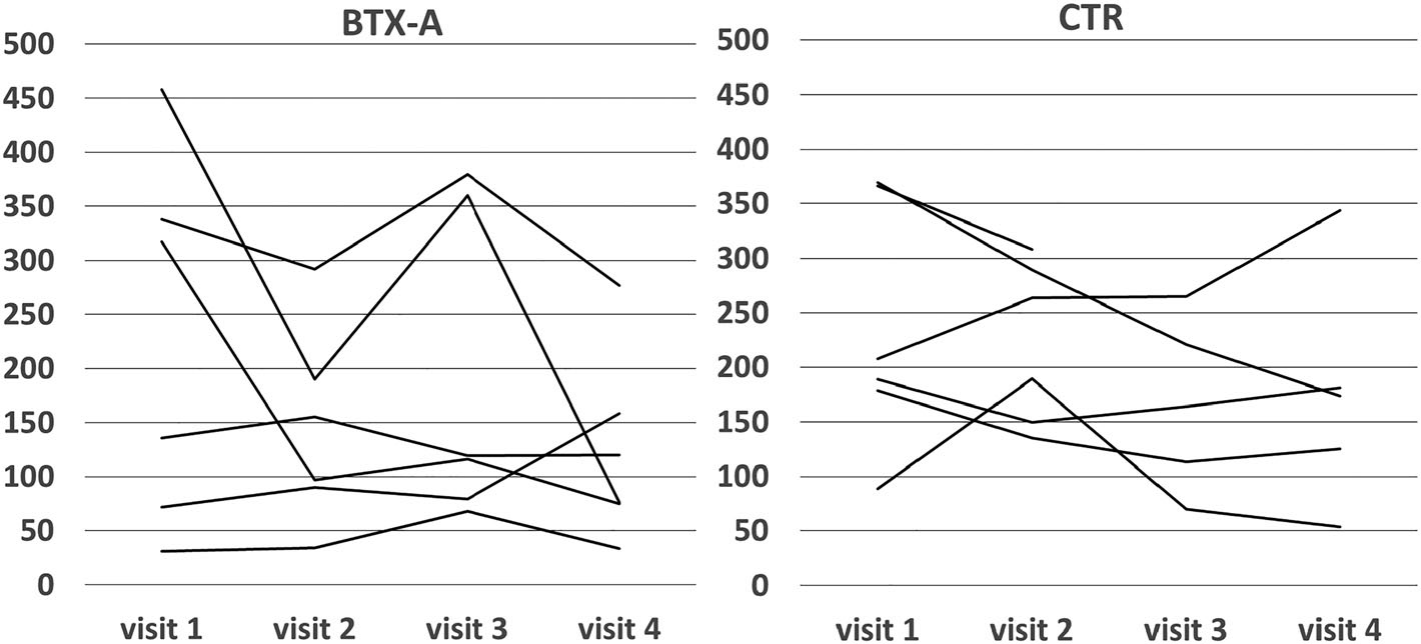
Individual data on bite force “as hard as you can” maximum voluntary contraction (N), at all visits for the 12 patients with BTX‐A or control (CTR; saline), injections respectively. Injection at Visits 1 and 3

**Figure 3:**
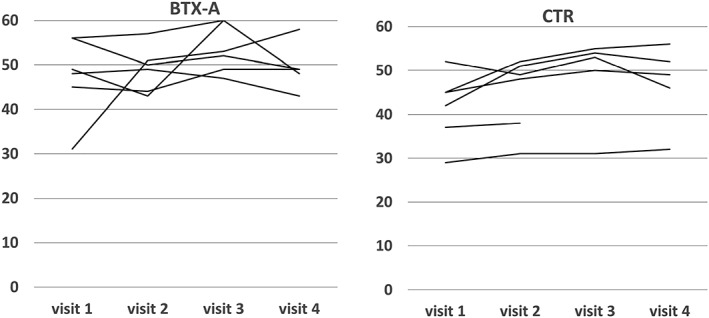
Individual data on GOHAI (scores 12–60), at all visits for the 12 patients with BTX‐A or control (CTR; saline), injections, respectively. Injection at Visits 1 and 3

The force at MVC and “as when chewing” in the active treatment arm declined substantially at group level (45% and 30%, respectively), but the reductions did not differ to a statistically significant degree from those in the control arm. Individual recordings of the MVC on all visits are given in Figure 2. Individual results for the GOHAI on all visits are given in Figure 3. The change in all 10 outcome variables from the first to the fourth visit among all patients is given in Table 3. The changes from the first to the fourth visit did not differ to any statistically significant degree for any outcome variable between patients who received BTX‐A or saline injections, respectively.

**Table 3:** Changes in all 10 outcome variables, mean (SD)/median (range) between the first and fourth visits for all patients (BTX‐A) and control (CTR; saline), respectively, and p values for differences

	Change from the first to fourth visit		
	BTX‐A	p ≤ .05	CTR
Bite force, N, “as hard as you can” (MVC)	−102.0 (175.4)/−38.4 (−381.8; 87.2) *n* = 6	NS	−31.2 (118.3)/−35.2 (−195.6; 135.6) *n* = 5
Bite force, N, ^“^as when chewing”	−34.3 (87.6)/−8.8 (−157.6; 79.9) *n* = 6	NS	28.8 (40.9)/33.7 (−38.4; 62.6) *n* = 5
Finger–thumb force, N	2.0 (2.7)/2.0 (−0.8; 4.8) *n* = 4	NS	−11.8 (36.5)/4.0 (−53.5; 14.1) *n* = 3
Chewing efficiency, mm	1.8 (3.5)/0.0 (0.0; 7.0) *n* = 4	NS	10.0 (21.2)/10.0 (−5.0; 25.0) *n* = 2
VAS#1, mm “prevalence bruxism”	−5.3 (27.9)/−12.0 (−44.0; 32.0) *n* = 6	NS	−18.5 (57.5)/−26.0 (−77.0; 55.0) *n* = 4
VAS#2, mm “pain in the jaws”	5.5 (37.7)/1.0 (−44.0; 73.0) *n* = 6	NS	16.2 (48.5)/1.0 (−26.0; 100.0) *n* = 5
VAS#3, mm “ability to chew”	−8.5 (48.7)/−7.0 (−81.0; 51.0) *n* = 6	NS	−0.50 (28.8)/−2.5 (−33.0; 36.0) *n* = 4
VAS#4, mm “ability to talk”	6.8(33.4)/3.0 (−55.0; 22.0) *n* = 4	NS	4.5 (19.7)/8.5 (−20.0; 21.0) *n* = 4
GOHAI, scores	3.6 (13.7)/−1.0 (−7.0; 27.0) *n* = 5	NS	4.4 (6.8)/4.0 (−6.0; 11.0) *n* = 5
TMI, scores	−0.01 (0.12)/0.00 (−0.16; 0.15) *n* = 6	NS	0.00 (0.11)/0.01 (−0.18; 0.10) *n* = 5

Abbreviations: BTX‐A, blocks with botulinum toxin type A; GOHAI, General Oral Health Assessment Index; MVC, maximum voluntary contraction; TMI, Temporomandibular Index; VAS, visual analog scale.

The number of responders, defined as at least a 30% decrease in MVC or a 30% improvement in GOHAI from the first to the fourth visit, respectively, were two in the active arm and two in the control arm for MVC and one in the active arm and two in the control arm for GOHAI.

Ten of all 11 still blinded patients who completed the study were positive about coming for a fifth visit for a BTX‐A injection 8 weeks after the study ended. After a further 3 months, seven of 11 patients (64%) were positive about continuing with further BTX‐A injections; four of the six patients formerly randomized to BTX‐A and three of the five patients formerly randomized to saline injections.

## 4 DISCUSSION

Not unexpectedly, the measured masticatory functions were stronger and more efficient among the healthy matched references and were also reported to be superior on average than those of the patients but not always to a statistically significant degree. No significant advantage of botulinum toxin injections, compared with control injections, could be demonstrated at group level for either objective or subjective outcome variables. The outcome variables in patients differed between subjects and over time, and the variation, range, and standard deviations were large at all registrations. Patients appeared to rate at least some of their oral abilities as far better than the objective measurements justified. These facts can partially explain the lack of statistically significant differences between treatment arms over time. However, 64% of the patients chose to continue with active injections after trying at least one BTX‐A injection. The majority of the patients therefore appeared to consider the benefits of BTX‐A injections to be better than any disadvantages. The findings are thus contradictory in this respect.

The most obvious weakness of the trial was that the number of participants was small. Moreover, it was terminated prematurely for several reasons. The results should therefore be considered as preliminary and admit only indicative deductions. The impact of active treatment on MVC was below that expected in some patients and not significantly different from that in the control arm. The power, analyzed post hoc with the patients' actual MVC change from the first to the fourth visit, was only 0.14. Based on the real outcome, the necessary number of patients would have been 75 in each treatment arm to reach a power of 0.80.

Because the burden of masticatory muscle hyperactivity, spasticity, and/or diurnal and/or nocturnal bruxism appears to be considerable among patients with “special needs,” it is essential to crucially investigate possible alleviating methods. The impact of bruxism on oral health‐related quality of life among young CP subjects is not negligible,(Abanto et al., 2014) but very few conventional treatments can be offered to these patients. The patient's own opinion of possible interventions has seldom been considered. A two‐group, parallel randomized controlled trial (RCT) design with objective and subjective outcome measurements was chosen for these reasons. An apparently diagnostically homogeneous group of patients, adults with CP and bruxism, was recruited for this intervention study. All the patients were noninstitutionalized with the ability to make decisions and communicate one way or another, which was considered important for ethical reasons. The intended target population is much larger, and, even if generalizability is difficult to estimate, extending the indications for BTX‐A injections to other groups with “special needs” was regarded as a desirable possibility.

The outcome variables were intended to reflect important, measurable aspects of oral function and abilities, pain and quality of oral health. Established, reliable methods evaluated force and chewing efficiency, and patient opinions were taken into account. The question “prevalence of bruxism” reflects only the patients' opinion and is not necessarily related to actual bruxism,(Raphael et al., 2015) but the patients fulfilled the criteria for probable bruxism according to international consensus statements.(Lobbezoo et al., 2013) A healthy, matched reference group was included not only for comparison but also to control the methods that were used. All the outcome variables in the reference group were similar over time, with one exception, indicating that the methods used were actually reliable when measured in a healthy, normal population.

Significant improvements in the active arm compared with the control arm could thus not be established at group level. The intention was to evaluate a group with similar problems, but the physical and mental status appeared to vary considerably among the patients. In spite of the fact that the patients had the same diagnosis, CP, the disparity of data between patients and over time in all variables was considerable and was perhaps the most striking finding. Patients were often satisfied with their oral ability, although an objective reduced capacity was registered. The associations between subjective and objective variables were less straightforward among patients than among references, indicating different reference frameworks. So objective reduced chewing efficiency did not necessarily correspond to a subjectively rated poor ability to chew among patients. Among references, increased chewing force was associated with increased pain ratings and increased chewing efficiency was associated with less pain. The findings are logical and differ from the associations found among the patients. The range of data among the references in “ability to chew” and “ability to talk” variables was only a fraction of that among the patients.

The BTX‐A injections were designed to act on two major muscles in the masticatory system. It is known that there is a needle effect in saline injections,(Tough, White, Cummings, Richards, & Campbell, 2009) but it is thought to act as a placebo. We found that botulinum toxin treatment was safe and well tolerated in this patient group. No complications following the injections were reported or observed. No further therapeutic arrangement was instituted in order to evaluate the particular effects of the injections. Including physiotherapy, for example, in the two groups might have improved effects and sustainability in both groups.

## 5 CONCLUSIONS

It can be established that the distributions of oral capacities vary greatly between subjects with CP and over time and are generally inferior to those in healthy subjects. Nevertheless, patients often rated their oral abilities as satisfactory, even when the corresponding objective recordings were less good. No ensured effect of active treatment on the chosen outcome variables at group level was observed, but continuing active treatment was requested by the majority of the patients. The evidence is thus not in favor of the benefit of BTX‐A injections but is inconclusive in certain respects. Only preliminary indicative conclusions are possible during the circumstances, and the research should be considered as a pilot study. Even more selective, homogeneous groups, based on preregistrations of important variables, and larger groups are probably necessary to reveal possible effects of botulinum toxin treatment on the masticatory muscles in patients with “special needs.”

## CONFLICT OF INTEREST

None of the authors have any conflict of interest.
